# Comparative study of liver injury induced by high-fat methionine- and choline-deficient diet in ICR mice originating from three different sources

**DOI:** 10.1186/s42826-019-0016-y

**Published:** 2019-08-17

**Authors:** Seunghyun Lee, Jae-Hwan Kwak, Sou Hyun Kim, Tae Bin Jeong, Seung Won Son, Joung-Hee Kim, Yong Lim, Joon-Yong Cho, Dae Youn Hwang, Kil Soo Kim, Young-Suk Jung

**Affiliations:** 1 College of Pharmacy, Pusan National University, Busan, South Korea; 2 College of Pharmacy, Brain Busan 21 Plus Program, Kyungsung University, Busan, South Korea; 3 Department of Clinical Laboratory Science, College of Nursing and Healthcare Science, Dong-Eui University, Busan, South Korea; 4 Exercise Biochemistry Laboratory, Korea National Sport University, Seoul, South Korea; 5 Department of Biomaterials Science, College of Natural Resources & Life Science/Life and Industry Convergence Research Institute, Pusan National University, Miryang, South Korea; 6 College of Veterinary Medicine, Kyungpook National University, Daegu, South Korea

**Keywords:** Non-alcoholic fatty liver disease, Liver injury, High-fat L-methionine- and choline-deficient diet, ICR mouse

## Abstract

Non-alcoholic fatty liver disease (NAFLD) is the leading cause of chronic liver disease worldwide. It is characterized by the accumulation of lipids without alcohol intake and often progresses to non-alcoholic steatohepatitis (NASH), liver fibrosis, and end-stage liver diseases such as cirrhosis or cancer. Although animal models have greatly contributed to the understanding of NAFLD, studies on the disease progression in humans are still limited. In this study, we used the recently reported high-fat L-methionine-defined and choline-deficient (HFMCD) diet to rapidly induce NASH and compared the responses to HFMCD in ICR mice from three different countries: Korea (supplied by the National Institute of Food and Drug Safety Evaluation), USA, and Japan during 6 weeks. Feeding HFMCD did not cause significant differences in weight gain in comparison with mice fed control diet. Relative weight of the liver increased gradually, while the relative weight of the kidneys remained unchanged. The parameters of liver injury (serum activities of alanine aminotransferase, aspartate aminotransferase, and lactate dehydrogenase) increased rapidly from 1 week and remained elevated for as long as 6 weeks. Histopathological analysis showed that the accumulation of hepatic lipids induced by HFMCD was prominent at 1 week after diet supplementation and increased further at 6 weeks. Inflammatory markers were significantly increased in a time-dependent manner by HFMCD. The mRNA levels of TNF-α and IL-6 were elevated approximately 15-fold relative to control diet and that of IL-1β was increased more than 20-folds at 6 week after the onset of HFMCD intake. In addition, mRNA expression of fibrosis markers such as α-SMA, TGFβ1, and Col1a1 were also significantly increased at 6 week. In summary, the responses of Korl:ICR mice by intake of HFMCD diet were similar to those of ICR mice from other sources, which suggests that Korl:ICR mice is also a useful resource to study the pathogenesis of diet-induced NAFLD.

## Introduction

Non-alcoholic fatty liver disease (NAFLD) is a liver metabolic disorder that does not involve alcohol intake. Importantly, over the years obesity rates have increased due to changes in lifestyle and food habits, and as a result NAFLD has become a common cause of chronic liver disease in many countries [1]. NAFLD includes a wide spectrum of liver diseases from simple steatosis to non-alcoholic steatohepatitis (NASH), fibrosis and cirrhosis, and ultimately hepatocellular carcinoma and liver failure [2]. Simple steatosis is usually not considered a serious condition. However, NASH can develop into cirrhosis or liver cancer, which may eventually be fatal [3]. Although many studies have been carried out, the pathological mechanisms of NAFLD remain to be elucidated and therapeutic drugs remain to be developed. The wide spectrum of NAFLDs makes it difficult to identify precise stage of disease, and the characteristics of very slowly progressive diseases are difficult to determine in clinical research [4]. Therefore, an animal model recapitulating human NAFLD can provide important information to determine the pathogenesis of the disease and to investigate the therapeutic effects of various drugs [5].

Animal models of NAFLD are largely classified as genetically engineered and nutritional (dietary) models according to etiology. In general, dietary induction of NAFLD in experimental animals is the preferred method to reproduce conditions observed in humans such as metabolic syndrome, whereas genetically engineered animals are used for detailed mechanistic studies [6]. Because the ICR mice have a low level of aggression and strong breeding ability, they are used worldwide for research on many diseases in diverse fields such as oncology, infections, and pharmacology [7].

The most widely used diet to induce NAFLD is the methionine- and choline-deficient (MCD) diet. It provides a very reproducible and efficient model to induce a severe NASH phenotype in a short period of administration such as 8 weeks [8]. Specifically, choline deficiency inhibits the synthesis of phosphatidylcholine, which is required for very low-density lipoprotein (VLDL) production, and is followed by lipid accumulation in the liver [9, 10]. The deficiency of the essential amino acid methionine decreases the biosynthesis of glutathione (GSH), the most potent antioxidant in the body, and leads to oxidative stress, which in turn contributes to liver damage [11]. However, MCD diet can cause serious weight loss, which is not usually observed in patients with NAFLD [12, 13]. Another well-studied dietary model is high-fat diet–induced NAFLD accompanied by obesity, although the diverse composition of such diets makes it difficult to compare studies from different research groups. Standard high-fat diets generally result in hepatic steatosis and do not induce significant NASH symptoms such as cell death, inflammation, or fibrosis even after feeding for more than 28 weeks [14]. A recent study introduced an improved mouse model to overcome the limitations of both MCD and high-fat diet [6]. The authors developed high-fat L-methionine- and choline-deficient (HFMCD) diet, composed of 60 kcal% fat, no added choline, and 0.1% methionine, by combining MCD with high-fat diet. This diet rapidly induced inflammatory response and fibrosis as well as steatosis in C57BL/6 J mice within 6 weeks without weight loss [6].

The Korl:ICR mice, which is the resident stock of the National Institute of Food and Drug Safety Evaluation (NIFDS), have been used for decades in terms of conducting Lot release project and more, in the NIFDS. According to the Nagoya Protocol, which describes a fair and equitable distribution of benefits arising from the use of genetic resources, securing national sovereignty over their resources is an important global issue. NIFDS identified biological characteristics of Korl:ICR compared with other ICR stocks to secure the indigenous data in 2017 [15]. Although there were no significant differences among the biological phenotypes of Korl:ICR and other ICR mice, phylogenetic analysis showed that the population stratification of the Korl:ICR was allocated different area from that of other ICR mice, suggesting that the Korl:ICR source colony could be a new stock in distinction from other ICR mice. In line with this, this study aimed to provide experimental results for securing Korl:ICR mice as Korea resource. Especially, we compared the response to the HFMCD diet of ICR mice from three different sources (NIFDS in Korea and suppliers in the USA and Japan) and evaluated the usefulness of the Korl:ICR mice in the research of pathogenesis of NAFLD and preclinical testing for drug development.

## Materials and methods

### Animals and treatment

Eight-week-old male ICR mice were obtained from three different sources. Korl:ICR mice were kindly provided by the Department of Laboratory and Animal Resources at the NIFDS (Cheongju, Korea). The other two groups of ICR mice were purchased from suppliers in the United States (A:ICR) and Japan (B:ICR). All animal experiments were approved by the Pusan National University Animal Experimentation and Use Committee (PNU-2018-1994). The basic conditions such as facility environment and diet were as described previously [16]. The mice were acclimated to 22 ± 2 °C and humidity of 55 ± 5% in the diet room with a 12-h light/dark cycle for 1 week prior to use. They were randomly divided into two groups fed different diets for 6 weeks: normal diet (control) or HFMCD composed of 60 kcal% fat, no added choline, and 0.1% methionine. The liver and kidneys were sampled at 1 and 6 weeks.

### Blood biochemical analysis

Blood samples were obtained from the abdominal aorta of each mouse; the sera were separated using a BD Microtainer Blood Collection Tube (BD Life Sciences, Franklin Lakes, NJ, USA) and used to measure activities of alanine aminotransferase (ALT), aspartate aminotransferase (AST), and lactate dehydrogenase (LDH). ALT and AST were measured using the protocols of Reitman and Frankel [17], and LDH was measured using a commercial kit purchased from Dogen (Seoul, Korea). The results were quantified with a spectrophotometer using a Multiskan GO reader (Thermo Scientific, Waltham, MA, USA).

### Histopathological analysis

The left lateral lobe of the liver was sliced and fixed with 4% paraformaldehyde. Tissues were embedded in paraffin, and a 5-μm section was stained with hematoxylin and eosin (H&E) to discriminate the nuclei and cytoplasm.

### RNA purification and quantitative RT-PCR

Quantitative RT-PCR was determined as reported previously [16]. Total RNA was isolated from the liver lysate using the Direct-zol RNA kit (Zymo Research, Orange, CA, USA). cDNA was synthesized with a iScript cDNA Synthesis system (Bio-Rad, Hercules, CA, USA). Quantitative RT-PCR was performed using the SensiFAST SYBR qPCR mix (Bioline, London, UK) according to the manufacturer’s protocol. The values of gene expression were normalized to those of *GAPDH*. Primer sequences are provided in Table 1.

**Table 1:** Primers used for quantitative RT-PCR

Genes	Primer Sequences	
TNF-α	F: GGCCTCTCTACCTTGTTGCC	R: CAGCCTGGTCACCAAATCAG
IL-6	F: TTGCCTTCTTGGGACTGATG	R: CCACGATTTCCCAGAGAACA
IL-1β	F: TTCACCATGGAATCCGTGTC	R: GTCTTGGCCGAGGACTAAGG
α-SMA	F: GCACCCAGCATGAAGATCAAG	R: TCTGCTGGAAGGTAGACAGCGAAG
TGFβ1	F: GCCCTGGATACCAACTATTGC	R: TGTTGGACAGCTGCTCCACCT
Col1a1	F: ACCTGTGTGTTCCCTACTCA	R: GACTGTTGCCTTCGCCTCTG
GAPDH	F: GTTGTCTCCTGCGACTTCA	R: GGTGGTCCAGGGTTTCTTA

### Statistical analysis

All data were expressed as mean ± standard deviation (SD). Statistical significance was determined using Student’s *t*-test, and *p* < 0.05 was considered significant.

## Results

### Effects of HFMCD diet on body weight change

**Fig. 1:**
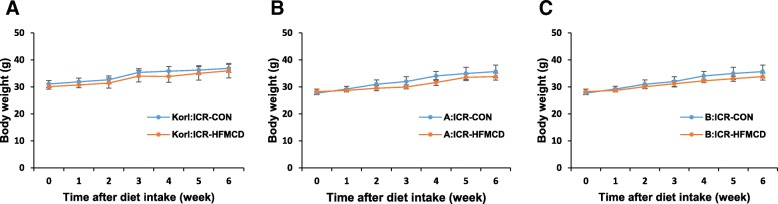
Effect of HFMCD on changes in body weight of Korl:ICR (**a**), A:ICR (**b**), and B:ICR (**c**) mice. ICR mice were fed control (CON) or high-fat L-methionine- and choline-deficient (HFMCD) diet composed of 60 kcal% fat, no added choline, and 0.1% methionine for 6 weeks. *n* = 8 per diet

High-fat diets cause obesity and insulin resistance, but 30 weeks are needed to establish NASH [14]. The MCD diet is often used to induce NASH by inducing hepatic steatosis and inflammation within 4 weeks, but it is characterized by severe weight loss [13]. Therefore, we attempted to induce NAFLD/NASH by using HFMCD diet. Body weight changes during 6 weeks showed no significant differences between control and HFMCD-fed mice (Fig. 1).

### Effects of HFMCD diet on changes in relative weights of liver and kidney

**Fig. 2:**
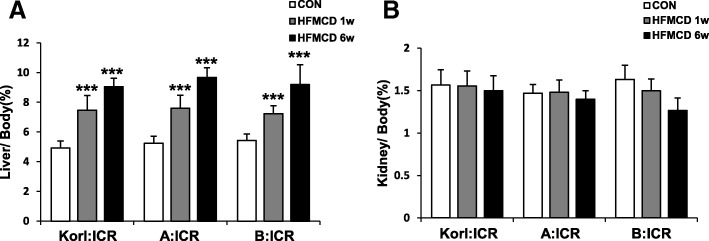
Effect of HFMCD diet for 1 and 6 weeks on the relative weights of the liver (**a**) and kidney (**b**) of mice from three different sources. *** Significantly different from the corresponding control mice (Student’s *t*-test, *P* < 0.001)

To examine the effect of feeding duration, we obtained samples at 1 and 6 weeks after HFMCD supplementation. The relative weight of the liver increased gradually (Fig. 2a) and at 6 weeks showed an approximately 2-fold increase in HFMCD-fed mice in comparison with control diet–fed mice. The relative weight of the kidneys remained unchanged during the experiment (Fig. 2b). The results were similar in all mice regardless of their origin.

### Effect of HFMCD diet on ALT, AST and LDH activities in serum

**Fig. 3:**
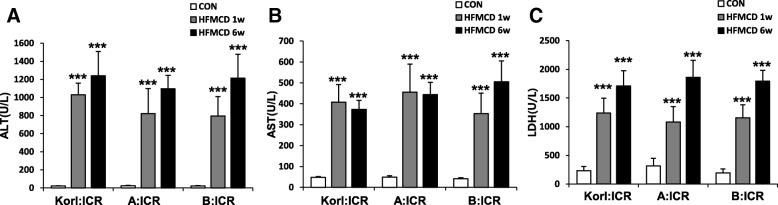
Effect of HFMCD diet for 1 and 6 weeks on the activities of (**a**) alanine aminotransferase (ALT) and (**b**) aspartate aminotransferase (AST), and (**c**) lactate dehydrogenase (LDH) in the serum of mice from three different sources. *** Significantly different from the corresponding control mice (Student’s *t*-test, *P* < 0.001)

Serum activities of ALT (Fig. 3a), AST (Fig. 3b), and LDH (Fig. 3c), which are indicators of liver injury, were significantly increased by HFMCD diet at 1 week and remained elevated at 6 weeks.

### Effects of HFMCD diet on histopathological changes in the liver

**Fig. 4:**
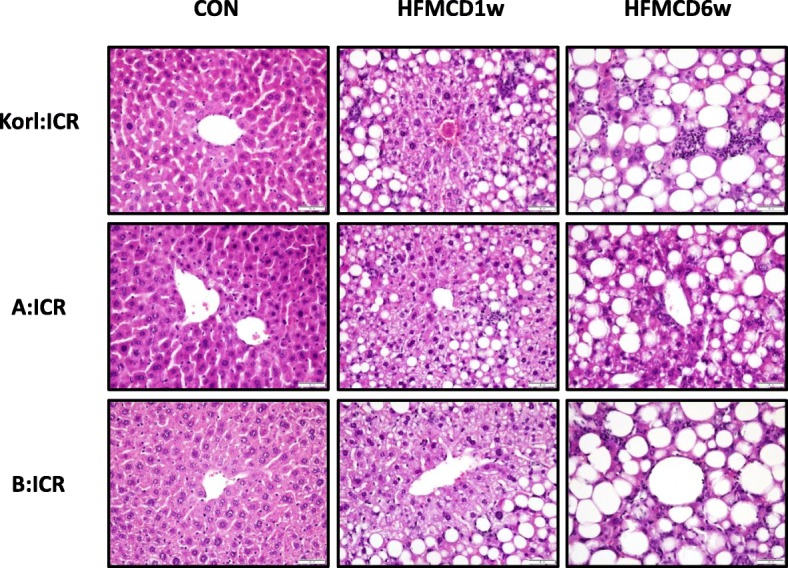
Effect of HFMCD diet for 1 and 6 weeks on lipid accumulation in the liver of mice from three different sources. Liver tissues were stained with H&E

We examined histopathological changes in the liver to find whether HFMCD induced the progression of NAFLD in a time-dependent manner. At 1 week, NAFLD induced both micro- and macro-vesicles, reflecting lipid accumulation in the liver; this effect was more severe at 6 weeks (Fig. 4). Macrovesicles occupied most of the liver, and neutrophil infiltration, which is an inflammatory reaction, was also observed in the liver of HFMCD-fed mice at 6 weeks. These results suggest that HFMCD induces progression from steatosis to NASH in a time-dependent manner within 6 weeks. Significance of the differences among mice of different origins was not examined.

### Induction of inflammatory response in the liver by HFMCD diet

**Fig. 5:**
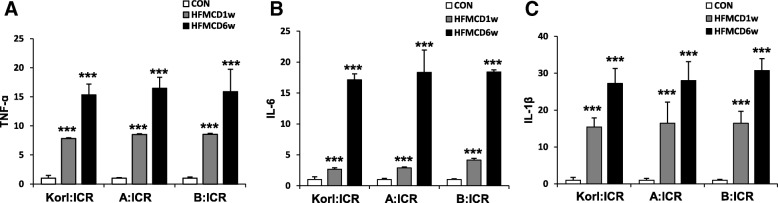
Effects of the HFMCD diet for 1 and 6 weeks on the mRNA levels of three inflammatory markers in mice from three different sources. *** Significantly different from the corresponding control mice (Student’s *t*-test, *P* < 0.001)

MCD diet increases the secretion of inflammatory cytokines and thereby induces liver inflammation, leading to NASH [18]. To confirm the induction of an inflammatory response by HFMCD suggested by neutrophil infiltration, we examined mRNA expression of TNF-α, IL-6, and IL-1β in the liver. All three cytokines showed time-dependent increases and were dramatically induced by HFMCD at 6 weeks after (Fig. 5). The levels of TNF-α (Fig. 5a) and IL-6 (Fig. 5b) transcripts were elevated approximately 15-fold and that of IL-1β (Fig. 5c) was increased more than 20-fold at 6 weeks after the intake of HFMCD.

### Expression of fibrosis markers in the liver by HFMCD diet

**Fig. 6:**
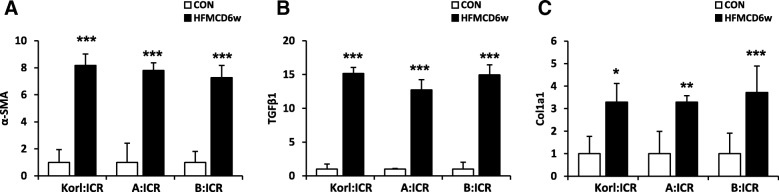
Effects of the HFMCD diet for 6 weeks on the mRNA expression level of the fibrosis markers of mice from three different sources. *, **, *** Significantly different from the corresponding control mice (Student's t-test, *P* < 0.05, 0.01, 0.001, respectively)

Fibrosis is considered a more advanced stage of NAFLD and we wondered whether intake of HFMCD for 6 weeks would affect liver fibrogenesis in ICR mice. The mRNA level of α-SMA and TGFβ1, markers for the activation of hepatic stellate cells, and collagen 1A1(Col1a1), one of ECM components, were significantly induced by HFMCD at 6 weeks after (Fig. 6). The transcript levels of α-SMA (Fig. 6a), TGFβ1 (Fig. 6b), and Col1a1 (Fig. 6c) were elevated approximately 8-, 15-, and 3-fold, respectively at 6 weeks after the intake of HFMCD.

## Discussion

NAFLD has become the most common cause of chronic liver disease worldwide as the incidences of obesity, diabetes, and metabolic syndrome have increased [19]. NAFLD includes a clinico-pathological spectrum of fatty liver diseases that occur in the absence of alcohol consumption [20]. The initial symptom is hepatic steatosis, which is considered to be a relatively benign liver injury. However, if the liver damage worsen, progression to NASH becomes faster and the mortality rate related to liver disease increases [21]. NASH is characterized by the presence of steatosis, inflammation, and hepatocellular death [22]. There is increasing evidence that NASH can cause progressive fibrosis, cirrhosis, and subsequently liver cancer [23].

The MCD diet has long been used to study NAFLD. MCD diet–fed animals show considerable lipid accumulation in the liver from 2 to 4 weeks, followed by inflammation and progression of fibrosis [24]. MCD diet reduces the VLDL secretion, increases fatty acid intake, induces inflammatory signaling, induces endoplasmic reticulum stress, and triggers lipid peroxidation [25]. It has the advantage of inducing NAFLD in a short period of time, but its disadvantage is a serious weight loss, which is not a common symptom in human NAFLD patients [26]. Therefore, HFMCD diet, which is a combination of high-fat and MCD diet, has been proposed to reproduce the pathological symptoms of NAFLD [6]. Importantly, this diet model mimics human disease, including many of the biochemical and histopathological features of NAFLD progression [27]. To date, some groups have reported differences among inbred mice strains in relation to their susceptibility to diet induced NAFLD and NASH [6, 28–31]. In particular, HFMCD diet induced more severe NASH phenotype with fibrosis in C57BL/6 mice compared with A/J mice [6], and long-term exposure to a HFD led to NASH in C57BL/6 J mice but not in A/J mice [31].

The ICR mice are outbreds that are non-consanguineous and heterogeneous, which brings them closer to representing natural populations. Currently, they are one of the most widely used experimental animals to study metabolic diseases such as obesity, diabetes, and NAFLD [32, 33]. Originally they were derived from Swiss mice developed at the Rockefeller Institute and are now produced in large quantities by a number of worldwide breeders [34]. The NIFDS in Korea has also established an ICR mouse stock called Korl:ICR and it was used for the last 50 years in the NIFDS [35]. This study aimed to compare the response of ICR mice from three different sources to HFMCD diet supply to ensure the usefulness of Korl:ICR in the research of NAFLD pathogenesis and preclinical drug development. HFMCD did not cause significant differences in body weight gain in comparison with control diet, but increased the relative weight of the liver in a time-dependent manner and dramatically increased the serum parameters of liver injury from 1 week after feeding. The accumulation of hepatic lipids induced by HFMCD was prominent from 1 week and was accompanied by significant inflammatory and fibrogenic responses at 6 week, as evidenced by neutrophil infiltration as well as accumulation of mRNA for pro-inflammatory cytokines and fibrosis markers. No significant differences in these responses were observed among the ICR mice from different sources.

## Conclusions

This study implicates that HFMCD model could be another option to overcome the disadvantage of the NAFLD model induced by the MCD or high-fat diet. We also found that the responses of Korl:ICR mice established by the NIFDS in Korea are similar to those of ICR mice from other sources, which suggests that it is a useful resource to study the pathogenesis of diet-induced NAFLD.

## Data Availability

The datasets used and/or analyzed in this study are available from the corresponding author on reasonable request.

## Abbreviations

ALT: Alanine aminotransferase
AST: Aspartate aminotransferase
GSH: Glutathione
H&E: Hematoxylin and eosin
HFMCD: High-fat L-methionine-defined and choline-deficient
LDH: Lactate dehydrogenase
NAFLD: Non-alcoholic fatty liver disease
NASH: Non-alcoholic steatohepatitis
NIFDS: National Institute of Food and Drug Safety Evaluation
VLDL: Very low-density lipoprotein
